# A Novel Variant in *CMAH* Is Associated with Blood Type AB in Ragdoll Cats

**DOI:** 10.1371/journal.pone.0154973

**Published:** 2016-05-12

**Authors:** Barbara Gandolfi, Robert A. Grahn, Nicholas A. Gustafson, Daniela Proverbio, Eva Spada, Badri Adhikari, Janling Cheng, Gordon Andrews, Leslie A. Lyons, Chris R. Helps

**Affiliations:** 1 Department of Veterinary Medicine and Surgery, College of Veterinary Medicine, University of Missouri—Columbia, Columbia, Missouri, 65211, United States of America; 2 Veterinary Genetics Laboratory, School of Veterinary Medicine, University of California Davis, Davis, California, 95616, United States of America; 3 Veterinary Transfusion Unit (REV), Department of Health, Animal Science and Food Safety (VESPA), University of Milan, 20133, Milan, Italy; 4 Department of Computer Science, Informatics Institute, University of Missouri–Columbia, Columbia, Missouri, 65211, United States of America; 5 Department of Diagnostic Service/Pathobiology, College of Veterinary Medicine, Kansas State University, Manhattan, Kansas, 66506, United States of America; 6 Langford Veterinary Services, University of Bristol, Bristol, BS40 5DU, United Kingdom; Faculty of Animal Sciences and Food Engineering, University of São Paulo, BRAZIL

## Abstract

The enzyme cytidine monophospho-N-acetylneuraminic acid hydroxylase is associated with the production of sialic acids on cat red blood cells. The cat has one major blood group with three serotypes; the most common blood type A being dominant to type B. A third rare blood type is known as AB and has an unclear mode of inheritance. Cat blood type antigens are defined, with N-glycolylneuraminic acid being associated with type A and N-acetylneuraminic acid with type B. Blood type AB is serologically characterized by agglutination using typing reagents directed against both A and B epitopes. While a genetic characterization of blood type B has been achieved, the rare type AB serotype remains genetically uncharacterized. A genome-wide association study in Ragdoll cats (22 cases and 15 controls) detected a significant association between blood type AB and SNPs on cat chromosome B2, with the most highly associated SNP being at position 4,487,432 near the candidate gene *cytidine monophospho-N-acetylneuraminic acid hydroxylase*. A novel variant, c.364C>T, was identified that is highly associated with blood type AB in Ragdoll cats and, to a lesser degree, with type AB in random bred cats. The newly identified variant is probably linked with blood type AB in Ragdoll cats, and is associated with the expression of both antigens (N-glycolylneuraminic acid and N-acetylneuraminic acid) on the red blood cell membrane. Other variants, not identified by this work, are likely to be associated with blood type AB in other breeds of cat.

## Introduction

Cytidine monophospho-N-acetylneuraminic acid hydroxylase (CMAH) is associated with the production of the sialic acids present on cat erythrocytes [1]. CMAH converts N-acetylneuraminic acid (NeuAc) to N-glycolylneuraminic acid (NeuGc) by substituting one of the hydrogen atoms in the methyl moiety with a carboxyl group [2,3]. Feline *CMAH* was the first gene shown to control blood types in non-primate mammals [4]. The gene is non-functional in humans [5,6], New World monkeys [7], birds and reptiles [8]. The ability to synthesize NeuGc was lost in hominids ~3 million years ago when an Alu element disrupted exon 6 of *CMAH* [5], and ~30 million years ago in the New World monkeys, through an inversion of exons 4 to 13 [7]. Loss of function of genes involved in membrane glycan synthesis is often an evolutionary defense mechanism to combat pathogen cell invasion [9,10], such as in the case of the malaria parasite *Plasmodium falciparum*, which prefers to bind to NeuGc [11,12]. By inactivating CMAH, the NeuAc precursor accumulates and NeuGc is absent on the erythrocyte cell surface, thus reducing parasite infection in humans and promoting a positive selection during evolution, which results in the enrichment of “resistant” blood type antigens.

The cat has one major blood group with three serotypes; the most common being blood type A that is dominant to type B. A third rare blood type is known as AB [13], and from breeding experiments has an unclear mode of inheritance due to apparently different inheritance patterns in different breeds [14]. Cat blood type antigens are defined, NeuGc is associated with type A and NeuAc with type B. Blood type AB is characterized by agglutination with antibodies against both NeuGc and NeuAc [1]. In a previous study, Bighignoli *et al*. (2007) demonstrated that the genomic structure of cat *CMAH* was similar to the homologous gene in other species [4]. The cat AB blood group system resulted from variant(s) in *CMAH* that prevented the conversion of NeuAc to NeuGc, hence type B to type A. Several variants, including SNPs upstream of the start codon, a deletion in the 5’ UTR of exon 1, and three variants in the coding region were concordant with A and B blood types in 18 different breeds of cat. Moreover, in the same study, the cats that tested positive for both NeuGc and NeuAc (type AB) could not be genetically differentiated from blood type A cats [4], hence blood type AB remained genetically uncharacterized. Note that the numbering in the original Bighignoli et *al*. paper is mis-aligned by 3 bases compared to the position in the feline coding sequence (CDS). Positions are updated in the current work, hence the original c.139G>A variant is actually at position c.142 of the *CMAH* CDS.

The frequencies of the different blood types vary among cat breeds and random bred populations with different origins. Several studies of cats from different continents confirmed blood type A to be the most common with a prevalence from 73% in Australia [13], 88.2% in China [15] to 95.3% in the United States West coast and 99.7% in United States Northeast [16]. Blood type B varies from 0.3% to 33% [16–19] and type AB is the rarest (0.2% to 20%) [13,20,21]. Blood type AB is common in Ragdolls with a frequency of 20% [22], Devon Rex (1.4%), British shorthair (1.6%) and particular random bred populations. In Japan, the proportion of blood type AB in the random bred population is 9.7% [23], 6.8% in Portugal [24], 5% in the UK [20], 2.3% in Brazil [25], 1.6% in Australia [26], and less than 0.2% in Canada [27] and the USA [14]. Whilst founder effects can explain the variation in frequency between breeds, and are probably the cause of the high percentage of type AB in Ragdolls, the variation between random bred populations strongly suggests an evolutionary enrichment of one blood type over another.

Cats possess naturally occurring antibodies against the blood antigen they lack [13,28,29]. Blood type B cats have strong anti-A alloantibodies [13,28] and blood type A cats have weak anti-B alloantibodies [28], while blood type AB cats have no alloantibodies since they express both antigens. Adverse reactions (such as vomiting, pyrexia, hemolysis, electrolyte disturbances, circulatory overload and urticarial) are observed when transfusing type A blood to a B recipient. A mild transfusion reaction can be observed if a type A recipient receives type B blood. However, if the type A cat has been previously exposed to type B blood, such as during pregnancy, serious transfusion reactions can occur, although these tend to be less severe and are less common than when type A blood is given to a type B cat. [16,29]. Blood type AB cats may develop a hemolytic reaction after a type B blood transfusion (due to strong anti-A alloantibodies in the blood donor) [14], hence transfusing type AB or type A blood is recommended. The lack of a feline universal blood donor requires that all cats must be blood typed and cross-matched before any transfusion and/or transplant. The characterization of blood type AB, and subsequent development of a genetic test, will increase the precision of genetic blood typing and help breeding programs. Moreover, once the type AB genetic test is developed, a substantial screening of blood types can be easily conducted and will allow the prevalence in different breeds and populations to be determined, offering insights into natural selection in different environmental conditions and resistance to pathogens associated with certain blood types. This study presents the genetic investigation of feline blood type AB in Ragdoll cats and confirms the power of the feline SNP array in detecting a phenotype–locus association. A genome-wide association study showed the blood type AB locus to be in close proximity to *CMAH*, which has previously been shown to control blood types A and B. Detailed investigation and screening of hundreds of cats supports the characterization of a novel feline blood type variant in Ragdoll cats.

## Results

### Genome-wide association study

The datasets supporting the results are included within additional supplementary files (including S1 and S2 Datasets).

**Fig 1:**
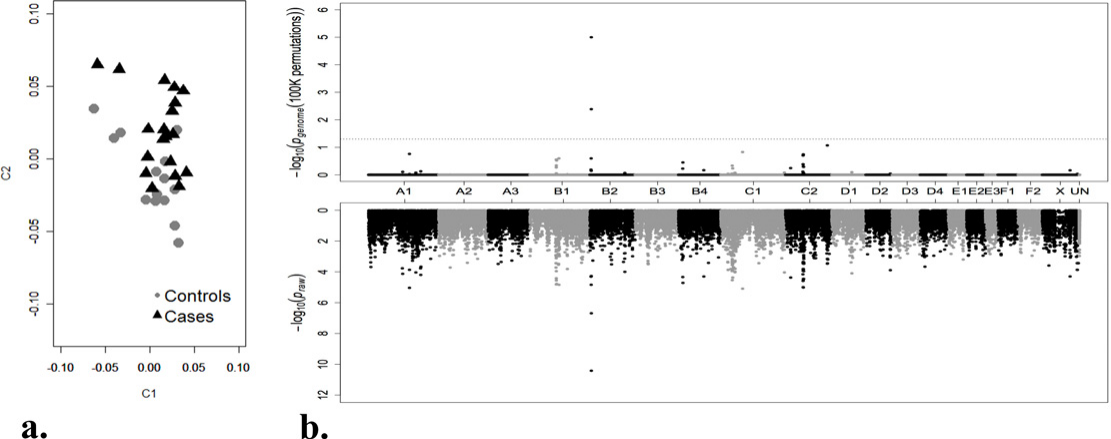
Multidimensional scaling and Manhattan plot summarizing the GWAS for Ragdoll cats with blood type AB. a. MDS showing the distribution of the Ragdoll cases and controls included in the analyses. Significant overlap of cases and controls suggests minimal substructuring in the dataset. b. The plot represents the *P*_*raw*_ (top) and *P*_*genome*_ (bottom) values of the SNPs included in the GWAS case–control analysis. The black dashed line (bottom) suggests the SNPs with genome-wide significance after permutation. Two SNPs on chromosome B2 (positions 4,487,432 and 4,449,822, respectively) remained significantly associated with the AB blood type phenotype.

The Ragdoll cases were selected by pedigree analysis to have minimal relationship to the controls, and 37 cats, including 22 cases (blood type AB) and 15 controls (blood type A, Ab or B), were submitted for SNP array genotyping (S1 Dataset, S2 Dataset). Testing for stratification revealed that four cats (one control and three cases) had a P̂ > 0.35, suggesting familiar relationships within the sample cohort. These samples were excluded from the genome-wide association study to reduce inflation of the results, leaving 19 cases and 14 controls for analysis. After the exclusion of the related samples, the genomic inflation (λ) was reduced from 1.72 to 1.50. Testing for stratification by multi-dimensional scaling (MDS) revealed that the cases and controls formed one main overlapping cluster, thus no additional samples were excluded (**Fig 1A**).

After initial quality control of the 62,897 SNPs on the array, 47,239 markers were included in the analysis; 15,557 were excluded for a minor allele frequency < 0.05 and 101 SNPs failed genotype frequency > 20% across all the samples. By analyzing 19 cases and 14 controls, a significant association was detected with SNPs on cat chromosome B2 (**Fig 1B**), and correcting *P*_*raw*_ values for multiple hypotheses testing by permutation, two adjacent SNPs on chromosome B2 at positions 4,487,432 and 4,449,822 respectively (**S1 Table**), were genome-wide significant (*P*_*genome*_ = 0.00001 and *P*_*genome*_ = 0.004) and remained significant after Bonferroni correction (1.8e^-6^ and 0.0097). The two SNPs are in linkage disequilibrium with the phenotype, the most common haplotype having a frequency of 79% in the cases and 10% in the controls, defining a short (~ 40 Kb) haplotype block. The haplotype block is in the intergenic region between two genes: the *leucine-rich repeat-containing protein 16A isoform 2* (*LRRC16A*) located ~50 Kb upstream of the haplotype block and *CMAH*, which is located ~40 Kb downstream. Since *CMAH* has been shown to control feline blood types A and B, this gene was further examined for cDNA and genomic variants that might be responsible for blood type AB.

### CMAH cDNA and genomic DNA comparison

Sequence data for *CMAH* cDNA was derived from six cats: four Ragdoll blood type AB, one random bred blood type AB, one random bred type B (serology B, genotype bb) and compared to the publicly available random bred type A (serology A, genotype AA) cDNA sequence (EF127684.1). Sequence analyses of the coding region identified nine cDNA variants, including five missense mutations. Four of the nine variants had previously been identified by Bighignoli *et al*. (2007), thus five *CMAH* variants (c.327A>C, c.364C>T, c.1158T>C, c.1269A>G, c.1398G>T) were novel to this study (**Table 1, S1 Fig**). Note that in **Table 1,** SNP positions within the *CMAH* CDS have been updated. Only the c.364C>T variant in exon 4, predicted *in silico* to change a nonpolar and hydrophobic amino acid (proline) to a polar and uncharged residue (serine) at codon 122, was present in the 5 type AB cats and absent in the type A and B cats. The c.364C>T variant was further investigated by genomic analysis in 280 cats with known blood type (see below).

**Table 1:** Comparison of *CMAH* cDNA variants in cats with blood types A, B and AB.

		Δ-53*	c.142*	c.268*	c.327	c.364	c.1158	c.1269	c.1398	c.1603*
Breed	Type	18bp del	G>A	T>A	A>C	C>T	T>C	A>G	G>T	G>A
Ragdoll #1, 2, 3, 4,	AB	NN	G	T	A	**T**	C	A	T	G
Random bred	AB	NN	G	T	A	**T**	C	A	T	G
Random bred	B	PP	A	A	C	**C**	C	A	G	A
EF127684.1	A	NN	G	T	A	**C**	T	G	K	G
AA change			V48M	Y90N	E109D	**P122S**	-	-	-	D535N

The (*) indicates SNPs that were previously reported by Bighignoli *et al*. (2007). The 18 bp indel is represented by N for not present and P for present. Note variant positions are updated (+3 bp) to match the *CMAH* CDS sequence in this work.

The c.327A>C variant was unique to the blood type B random bred cat. Two of the three synonymous mutations (c.1158T>C, c.1269A>G) were found exclusively in the blood type A random bred (from reference sequence EF127684.1), while c.1398G>T was found in the type A and B random bred cats and was not present in the type AB phenotype cats (**Table 1**).

### CMAH variant genotyping

The newly identified c.364C>T variant in exon 4 was genotyped in 280 cats with known blood types defined by serology and was found in 89 of 115 type AB cats with at least one allele coding for blood type A. The variant was only detected in Ragdoll and random bred cats. Four type AB Ragdoll and 13 type AB random bred cats were wild-type for c.364C>T. The variant was not associated with blood type AB in Devon rex (n = 8) and British shorthair (n = 1) cats and was never found in blood type A or B cats (n = 164) (**Table 2**).

**Table 2:** Domestic cats with known blood type serology genotyped for the c.364C>T variant in *CMAH* exon 4.

				Genotype c.364C>T		
Breed	Blood type	#	C/C	C/T	T/T	N/A
American shorthair	A	1	1	0	0	0
Birman	A	29	29	0	0	0
	B	11	11	0	0	0
British	A	8	8	0	0	0
shorthair	B	9	9	0	0	0
	AB	1	1	0	0	0
Burmese	A	2	2	0	0	0
Devon rex	A	8	8	0	0	0
	B	24	24	0	0	0
	AB	8	8	0	0	0
LaPerm	A	1	1	0	0	0
Maine coon	A	1	1	0	0	0
Persian	A	1	1	0	0	0
	B	1	1	0	0	0
Ragdoll	A	28	28	0	0	0
	B	1	1	0	0	0
	AB	58	4	31	22	1
Random bred	A	18	18	0	0	0
	B	6	6	0	0	0
	AB	49	13	33	3	0
Siamese	A	2	2	0	0	0
Siberian	A	3	3	0	0	0
	B	2	2	0	0	0
Singapura	A	5	5	0	0	0
Snowshoe	A	1	1	0	0	0
Somali	A	1	1	0	0	0
Tiffanie	A	1	1	0	0	0
Total		280	190	64	25	1

Other *CMAH* variants were also investigated in the 280 cats: the 18 bp deletion within the *CMAH* 5’UTR, the exon 2 c.142G>A and the exon 2 c.139C>T (LVS cats only) (**S2 Table**). The exon 2 c.142G>A and c.139C>T variants were originally numbered c.139G>A and c.136C>T in Bighignoli *et al*. (2007) and Tasker *et al*. (2014), respectively. The 18 bp deletion and c.142G>A variant, previously associated with feline blood type B, were found on the feline B allele, confirming the findings from the Bighignoli *et al*. (2007) study. However, nine cats with known blood type A or B serology showed a discrepancy between the deletion and the c.142G>A variant. In seven cats, the c.142G>A was associated with the blood type confirmed by serology while the deletion was discordant. For two cats, both variants were discordant and suggested a serology mistype. For one cat, the deletion was concordant with the blood type, suggesting the missense mutation (c.142G>A) as more likely causal for blood type B, although an error in blood serotyping cannot be excluded.

To determine the allele frequency of the c.364C>T variant, 786 cats from many breeds with unknown blood type were genotyped for the newly identified variant, as well as the 5’ UTR deletion and the c.142G>A missense mutation. The c.364C>T variant was only detected in the Ragdoll, random bred and Cornish rex cats; the frequency of the T allele in these breeds was 8.8%, 10% and 2.4%, respectively (**Table 3**). Cats with the c.364C>T variant were genotyped as AB/AB, AB/A or AB/b. In the 786 cats tested, 53 (6.7%) showed a discrepancy between the 5’ UTR deletion and the c.142G>A variant (**S4 Table**).

**Table 3:** Frequency of the *CMAH* c.364C>T polymorphism in different cat breeds with unknown serology.

			c.364C>T		
Breed	#	C/C	C/T	T/T	T allele frequency
Abyssinian	42	42	0	0	0
Bengal	13	13	0	0	0
Birman	43	43	0	0	0
British long / shorthair	100	100	0	0	0
Cornish rex	21	20	1	0	**2.4**
Devon rex	58	58	0	0	0
Exotic SH	25	25	0	0	0
Maine coon	21	21	0	0	0
Ragamuffin	9	9	0	0	0
Ragdoll	266	220	45	1	**8.8**
Random bred	15	12	3	0	**10**
Siberian	38	38	0	0	0
Somali	24	24	0	0	0
Sphynx	16	16	0	0	0
Unknown	37	33	4	0	**5.4**
21 other breeds (n<9)	58	58	0	0	0
**Total**	786	732	53	1	**3.5**

Since the c.364C>T variant was not concordant in all blood type AB cats, genomic analysis of *CMAH* was conducted on two type AB Devon rex, one type AB British shorthair, one type AB random bred cat and one type A random bred cat that were wild-type for the c.364C>T variant. Twelve variants were detected: seven in the coding region, one in the 5’ UTR and four in intronic regions. Five novel variants were identified, four located in introns (I3 c.307+22A>G, I9 c.1112+29T>G, I9 c.1112+66G>A, I9 c.1113-70G>A) and one silent mutation in the coding region, (c.884G>A). Only the variant localized 70 bp upstream of exon 10 (I9 c.1113-70G>A), was concordant with blood type AB, but was not predicted to disrupt any conserved splice site regions and was not investigated in detail (**S3 Table**).

### Computer modeling of feline CMAH protein structure and c.142G>A (V48M)

Templates with known tertiary structure were identified for two regions of the CMAH protein sequence (from residues 21 to 94 and from 207 to 294) using PSI-BLAST [30] with an e-value threshold of 0.005. For the first region (residues 21 to 94), the top template is a Rieske iron-sulfur protein 2PQZ, which covers the first variant site of interest (c.142G>A, V48M). Iron-sulfur [Fe-S] clusters are ubiquitous and required to sustain central life processes. Fe-S clusters participate in electron transfer, substrate binding/activation, gene expression regulation and enzyme activity [31]. The template (**S2 Fig**) shows that the metal-ion binding site is spatially distant from the V48M variant. The other template covering the second region of the CMAH sequence (residues 207 to 294, **S2 Fig**) is part of the complex of a large ribosomal subunit from *Deinococus radiodurans* (3JQ4) with a zinc binding site. The 3-D protein structure surrounding the novel P122S variant could not be modeled due to the lack of an available 3-D template.

## Discussion

Sialic acids are a family of sugars that share a nine carbon backbone and are typically found on cell surfaces and glycan molecules. About 40 sialic acids are documented with the most common being NeuAc, which is a substrate for the synthesis of NeuGc by CMAH. These two sialic acids are the major determinants of feline blood types A and B. A third, rare and genetically uncharacterized blood type (AB) also exists with differing frequencies amongst cat populations. The A/B blood group system is clinically important in cats because mismatched breeding and transfusions can cause life threatening hemolytic reactions without prior sensitization [14]. Therefore, a complete genetic characterization of all feline blood types is crucial so that genetic testing can accurately report an individual’s blood type.

Recent advances in feline genomics, such as a new genome assembly [32] and the availability of a high density SNP array chip, have accelerated the discovery of several genetic diseases [33–35] and phenotypic traits [36,37] in the domestic cat. The Ragdoll is a relatively new breed with western origins (California, USA) [38,39] and characterized by point coloration and long hair [40,41]. Ragdolls are not significantly related to any other breed. In a previous study, Alhaddad *et al*. (2013) [42] reported that a recently developed breed might have longer haplotype blocks, enabling genome-wide association studies with fewer individuals [36,37]. Strong selection for fewer aesthetic traits and a breeding program dependent on very few founders results in high levels of LD and long haplotype blocks, characteristic of identity by descent traits. In this study, using only 33 individuals, an association of blood type AB with a variant on cat chromosome B2 in the Ragdoll breed was detected. Likely, during the Ragdoll breed development, a cat with the AB phenotype was extensively used in the breeding program, introducing the variant into the breed with a high frequency.

Due to the overall low prevalence of blood type AB, it was unclear whether *CMAH* was the locus associated with the AB blood type phenotype, or whether there were different loci involved, in fact, the possibility of cis-acting alleles was previously suggested [13]. Griot-Wenk *et al*. [14] presented strong evidence that type AB was allelic, but the presence of a third allele could not explain blood type inheritance in British shorthair and Somali cats, additionally, the inheritance of blood type AB was never studied in Ragdoll cats, hence the need for an association study. The GWAS indicated that the locus influencing the rare blood type AB resided on cat chromosome B2, within one of two genes: *LRRC16A* and *CMAH*. Since *LRRC16A* is responsible for the inhibition of actin filament capping, which results in enhancing actin polymerization [43], *CMAH* remained the top candidate gene for the blood type AB phenotype, given that the gene is the major locus controlling blood types A and B in cats [4]. Although Bighignoli *et al*. [7] included a type AB Siamese cat in their study they were not able to identify a causal variant for blood type AB. Furthermore, Ragdoll cats were not included in the previous study and their *CMAH* CDS was only determined in the current study. In this study, a novel variant in *CMAH* exon 4 (c.364C>T) that causes a proline to serine amino acid substitution at position 122 (p.122P>S) was identified. Proline is the only amino acid where the side chain is connected to the protein backbone twice and is often found in very tight turns in protein structures. The proline side chain is non-reactive and is rarely involved in a protein’s active or binding sites, while serine is quite common in protein functional centers and is fairly reactive. Prolines are considered important in protein structure, hence this substitution might be associated with an abnormal protein conformation, resulting in a partial or aberrant enzyme function. The newly identified variant is highly associated with blood type AB in Ragdoll cats, with only four of the 58 blood type AB Ragdoll cats being homozygous for the wild-type exon 4 allele. In these discordant cats, another variant at a second locus or at the same locus in the non-coding sequence may be the cause of their blood serotype or it could be due to a database or sampling error. Alternatively, their blood phenotype could be incorrect due to inaccurate serotyping or a change in blood type serology due to sickness. A previous study showed that humans absorb a portion of ingested NeuGc and incorporate a small amount into newly synthesized glycoconjugates [44,45]. Julien et al. [46] and Mortezai et al. [47] demonstrated that the presence of NeuGc and NeuAc improves local inflammatory responses, hence causing a temporary variation in blood serology results.

Feline blood typing can be commercially performed using several methods including a card that uses monoclonal antibodies [48], a matrix gel column containing serum or lectin [49], a slide and tube assay that uses lectin and anti-A sera [50] and a commonly used typing kit based on immunochromatographic diffusion of red blood cells [51], which has recently been shown to outperform the card method with 100% specificity and sensitivity in anemic and non-anemic cats [52]. Seth et *al*. (2011) [51] compared feline blood typing methods and showed that agreement between the methods varied from 99.4% to 91.4% when compared to the tube assay. Therefore, potentially the four discordant Ragdoll cats submitted as blood type AB, were actually type A or B [51]. In the domestic cat population, the newly identified variant was found in 73% of blood type AB cats, and was absent in type AB cats from the British shorthair and Devon rex breeds. Direct sequencing of the *CMAH* CDS in Devon rex and British shorthair cats with blood type AB did not suggest any previously undetected missense or splice site disrupting variants, suggesting blood type AB is controlled by a different locus, or a variant in the same locus in a non-coding region, in these breeds.

Both NeuGc and NeuAc sialic acids appear to be present at low levels on the red cell surface of blood type AB cats [1]. The presence of both antigens on type AB cats’ erythrocytes can be explained by a reduction in CMAH activity. Several studies showed that the amount of NeuGc incorporated on the cell membrane is regulated by the level of hydroxylase activity [53,54], hence the newly identified c.364C>T variant may reduce CMAH activity. Tasker *et al*. [20] reported a previously unknown c.136C>T (numbered c.139C>T herein) variant associated with feline blood type B; potentially every novel variant that knocks-out CMAH activity could be associated with blood type B. By comparison, all variants that reduce CMAH activity may be associated with blood type AB, suggesting the possibility of more, as yet unidentified, variants associated with feline blood types and the need for further molecular investigation. A three-dimensional prediction of the effect of the c.142G>A (V48M) missense mutation associated with blood type B suggests that CMAH is still capable of binding the substrate (since the substitution does not disrupt the [Fe-S] domain) but, since NeuGc is not synthesized, the enzyme activity must be compromised by the variant [1]. Unfortunately, no insights were gained from the protein structure prediction on the effects of the substitution associated with blood type AB, mainly due to the lack of a viable protein template for the region.

The AB serotype is allelic to A and B in cats, and the variant associated with type AB resides on the enzymatically active type A allelic background, and is likely associated with a reduction of CMAH activity and the presence of both sialic acids on the red cell surface. A three allele system is proposed: *A* > *a*^*AB*^ > *b*, where a type A cat may have *AA*, *Aa*^*AB*^ or *Ab* genotype. The type B cats are always homozygous *bb*. Possible genotypes / phenotypes (serotypes) would be *AA* (type A); *Aa*^*AB*^ (type A); *Ab* (type A); *a*^*AB*^*b* (type AB); *a*^*AB*^*a*^*AB*^ (type AB), and *bb* (type B). Whilst the majority of blood type AB cats in the study were *a*^*AB*^*b* or *a*^*AB*^*a*^*AB*^ a significant number (n = 27) were genotype *Aa*^*AB*^, and would be predicted to be blood type A. This apparent discordancy could be explained by a second, yet to be identified, type AB variant on the A allele, and is not an unexpected finding since other breeds with blood type AB were shown not to have the c.364C>T variant. An in-depth molecular investigation of samples with discrepancies between genotype (c.142G>A, c.364C>T) and blood phenotype is needed in future studies to detect other alleles involved in feline blood types.

## Conclusion

This study identified a variant associated with the rare feline AB blood type in Ragdoll cats. A genome-wide association study suggested *CMAH* as a candidate gene and a newly identified c.364C>T missense mutation is probably linked with the feline AB blood type. Moreover, the variant is likely associated with a reduction of CMAH enzyme activity and the resultant expression of both antigens (NeuGc and NeuAc) on the red cell membrane. Since blood type AB cats from other breeds did not have the Ragdoll type AB variant, at least one other variant must exist that also results in a type AB phenotype.

## Methods

### Ethics statement

This study was approved by the Animal Care and Use Committee (ACUC) of the University of Davis (protocol # 16991) and the University of Missouri (protocol # 7808). Italian samples were collected with the informed consent of owners during routine wellness visits. Several samples were acquired by specialist in the field, such as a veterinarian, or voluntarily donated by owners and breeders with consent to use the specimens for research purposes.

### Sample collection

European and USA cat owners and breeders voluntarily donated EDTA anti-coagulated whole blood or buccal swabs from blood type A, B and AB cats (n = 134). Additionally, 138 DNA samples from cats blood typed by hemagglutination and alloantibody assays [13] were also provided by several laboratory services: (1) Oy Triniini Company, Helsinki, Finland, (2) Langford Veterinary Services (LVS), Bristol, UK, (3) Department of Diagnostic Medicine/Pathobiology, College of Veterinary Medicine, Kansas State University (4) and the University of Milan, Italy. The complete sample set (N = 280) included 110 type A samples, 54 type B and 116 type AB. The sample set included 73 random bred cats and 15 breeds (87 Ragdoll, 40 Birman, 40 Devon Rex, 18 British shorthair and 22 cats from 11 other breeds, **Table 2**).

The Veterinary Genetic Laboratory (VGL) at the University of California, Davis, genotyped 786 samples with unknown blood serology/phenotypes, from several different breeds and random bred cats (**S4 Table**).

DNA from 37 Ragdolls (22 type AB and 15 type A, Ab or B) were submitted to Geneseek Inc. (Lincoln, NE) for genotyping analysis on the array. DNA extraction and quality control was conducted as previously described [33]. Supplementary files contain the genotyping data (S1 Dataset) and the location of each SNP typed on the array (S2 Dataset). Blood from four Ragdoll type AB, one random bred type AB and a random bred type B was also collected in PAXgene^TM^ (Qiagen, Valencia, CA) tubes for RNA extraction.

### Availability of Data and Materials

The supporting file section contains the raw genotyping data for the 37 Ragdolls included in this study (S1 Dataset) and the genomic location of each variant (S2 Dataset) typed on the Illumina Infinium Feline 63K iSelect DNA array (Illumina, Inc., San Diego, CA).

### Case-control genome wide association analysis

Initially, genomic DNA from 22 type AB Ragdolls (cases) and 15 type A, Ab or B Ragdolls (controls) was genotyped on the Illumina Infinium Feline 63K iSelect DNA array. SNP genotype data analysis was performed with PLINK [55] and subjected to standard quality control. SNPs were excluded for poor genotyping rates (< 80%), low minor allele frequencies (< 0.05%) and individuals were excluded for low genotyping success (< 80%). Inflation of p-values was evaluated by calculating the genomic inflation factor (λ). To reduce genomic inflation, individuals with a proportion of IBD > 0.35 were excluded from the downstream analyses. A classic multi-dimensional scaling (MDS) with 2-dimensions was performed in PLINK to evaluate population substructure within cases and controls. The case-control association analysis was performed and corrected with 100,000 t-max permutations (**Fig 1**). T-max calculated p-values were considered genome-wide significant at p < 0.05. After each correction, the *P* value of the highly associated SNPs was evaluated and reported in **S1 Table**. All genomic positions are reported in accordance with the Felis_catus 6.2/felCat5 build of the feline genome [32].

### CMAH gene and CDS analysis

Total RNA was extracted using the PAXgene™ kit (Qiagen) from whole blood from six cats including four Ragdoll type AB, one random bred type AB and one random bred type B. Complementary DNA was synthesized using SuperScript III (Invitrogen, Carlsband, CA) by reverse transcription of 1 μg of total RNA with gene specific primers (CMAH-1R, CMAH-2R, CMAH-3R, CMAH-4R) and oligo dT to obtain UTRs and CDS **(S5 Table)**. Each cDNA sample served as template for PCR using 1 μM primers combined as follow: CMAH-1F + CMAH-1R, CMAH-2F + CMAH-2R, CMAH-3F + CMAH-3R, CMAH-4F + CMAH-4R, CMAH-3UTR+ polyT. The PCR conditions were: 1.5 mM MgCl_2_, 1X Buffer, 0.1 U of AmpliTaq Gold DNA polymerase (Thermo Fisher Scientific, Waltham, MA) and 2 μl of cDNA in a total volume of 20 μl. The PCR was: initial denaturation at 94°C for 4 min followed by 40 cycles at: 94°C for 30 sec, 62°C for 30 sec, 72°C for 1 min and a final extension at 72°C for 20 min. The PCR products were purified and sequenced as previously described [4]. The 5’ UTR was obtained as previously described using the gene specific primer CMAH-5UTR (**S5 Table)**, the PCR conditions included an initial denaturation of 5 min followed by 40 cycles of 30 sec denaturation at 94°C, annealing for 30 sec at 56°C and extension at 72°C for 30 sec, followed by 10 min extension at 72°C.

Genomic analysis of *CMAH* was conducted on four type AB cats (one random bred, one British shorthair, two Devon rex) and one type A random bred cat. *CMAH* CDS sequence is available and can be found on GeneScaffold ENSFCAG00000002804 (ensemble.org). Primers for the amplification of the coding region of *CMAH* were designed in both UTRs and intronic regions, flanking the exons. Primers were tested for efficient product amplification on a DNA Engine Gradient Cycler (MJ Research, GMI, Ramsey, MN) and the final PCR conditions were 1.5 mM MgCl_2_, 1X Denville Buffer, 1 uM primer, 0.2 U Taq and 10 ng DNA. Thermal cycling conditions were as follows: initial denaturation at 94°C for 4 min, followed by 40 cycles of: 94°C for 30 sec, 60°C for 30 sec, 72°C for 45 sec and final extension at 72°C for 20 min. The PCR products were purified with ExoSap (USB, Cleveland, OH) following the manufacturer’s recommendations and directly sequenced using the BigDye terminator Sequencing Kit v3.1 (Applied Biosystems, Foster City, CA). Products were purified with Illustra Sephadex G-50 (Ge Healthcare, Piscataway, NJ) according to manufacturer’s recommendations. Sequence data was analyzed with Sequencer 5.1 (GeneCodes, Ann Arbor, MI, USA).

### Variant analysis of feline CMAH

Seven *CMAH* variants were previously described as concordant with blood type B [4]. Of these, two upstream SNPs (c.1-371C>T and c.1-217G>A), two missense mutations (c.268T>A and c.1603G>A) and one silent variant (c.1269G>A) have been shown not to be associated with blood type B (data not shown) or with a low impact on the protein and not prioritized in the genetic testing. The remaining two variants, a 18 bp deletion (c.1-53delGTCGAAGCCAACGAGCAA) in the 5’UTR and the c.142G>A are predicted to have an impact on the cytidine monophospho-N-acetylneuraminic acid hydroxylase activity and are in linkage disequilibrium with blood type B, and were hence genotyped when possible in all samples included in the study. The c.142G>A variant associated with blood type B [4] was genotyped by direct sequencing using the primer set CMAH-2F and CMAH-2R (**S5 Table**). The c.142G>A variant is included in the 62,897 SNPs tested on the Illumina array, hence data for the locus was obtained for all GWAS samples by exporting the genotyping results. The 18 bp deletion was genotyped as a size variant by direct PCR using the FAM-CMAHdelF labelled primer and the CMAHdelR primer and electrophoretically separated on an ABI DNA analyzer. The predicted size of the wild-type allele was 319 bp, and 301 bp for the B allele and verified using the program STRand [56]. The PCR conditions were as described: 2 min denaturation at 95°C, followed by 40 cycles at 95°C for 30 sec, 58°C for 30 sec and 72°C for 45 sec, followed by a 10 min extension at 72°C. The newly identified c.364C>T variant was genotyped by direct sequencing using the genomic primers CMAH4F and CMAH4R, and sequenced as previously described in the genomic analysis section (**S5 Table**).

At the VGL, an allele specific assay was developed for the detection of c.364C>T. Each PCR contained 3μl of template DNA isolated as previously described [57]. Thermal cycling consisted of: 95°C 3 min, 85°C 5 min, 33 cycles of 95°C 45 sec, 60°C 30 sec, 72°C 30 sec, followed by 1 cycle of 72°C for 15 min and a 10°C hold. Each 25 μl reaction contained 1μM of each primer (the forward FAM-364, the wild-type reverse 364wtR and the affected reverse 364affectedR), 1X reaction buffer (Denville Scientific, Inc., South Plainfield, NJ), 2.5mM MgCl_2_, 1.0mM dNTP, 0.02μl DMSO (Fisher Scientific) and 1.0U Choice *Taq* DNA polymerase (Denville Scientific Inc.). Template specificity was increased by incorporating a sequence mismatch in the reverse primer (lower-case letter) and the addition of 7 mismatched bases (underlined) created a visually detectable product size variant. To decrease background, template DNA and primers were initially denatured for 3 min at 95°C and the remaining 15 μl of reaction mix was added during the 85°C pre-cycling soak. PCR amplicons were visualized on an ABI3730 DNA analyzer (Applied Biosystems) and analyzed using STRand software [56].

At LVS, pyrosequencing was used to detect c.142G>A, c.139C>T and c.364C>T. Each PCR contained GoTaq Master Mix (Promega, UK), 0.2μM each primer (exon 2 primers for c.142G>A, c.139C>T and exon 4 primers for c.364C>T) (**S5 Table**) and 2μl of template DNA. Thermal cycling consisted of 95°C for 2 min followed by 36 cycles of 95°C for 20 sec and 58°C for 40 sec, with a final 10°C hold. Amplicons were purified and subjected to pyrosequencing using specific sequencing primers (**S5 Table**) and PyroGold reagents as described by the manufacturer (Qiagen, UK) on a PyroMark Q24 pyrosequencer (Qiagen).

### CMAH computer modelling

A 3-dimensional computer model for the wild-type cat CMAH protein (EF127684.1) was built following a template based modeling procedure [58]. Specifically, PSI-BLAST [30] was used to search the sequence of CMAH against the sequences in the Protein Data Bank (PDB) [59] to find homologous proteins with known tertiary structures. The homologous protein matches were ranked by the significance of sequence similarity (e-value) and the percentage of sequence identity and the top three templates analyzed. Finally, using the 3-dimensional structure of the top ranked protein as a template in conjunction with the sequence alignment, a 3-dimensional structure for the wild-type CMAH protein was built, for the regions for which templates were found, using Modeller [60]. The structural model of the wild-type protein was then used as a template to construct the structural model of the mutated protein.

## Supporting Information

S1 Dataset Genotyping raw data. (PED)

S2 Dataset Genotyping map coordinates. (MAP)

S1 Fig Chromatograms showing the variants in *CMAH*. **a.** The c.142G>A variant associated with blood type B (red rectangle), all possible genotypes are shown. Three bases upstream, in the black rectangle, is shown the c.139C>T variant. **b.** The c.364C>T variant associated with blood type AB (red rectangle). All possible genotypes are shown.
(PDF)

S2 Fig Three-dimensional model and templates found by PSI-BLAST for the feline sequence against the PDB database. (**top**) The two domains found in the database used to partially modulate the structure of the protein from position 21 to position 94 and from position 192 to position 288 of the CMAH protein. The relative position of the V48M variant associated with blood type B is shown in red in the 2QPZ template and the four zinc-binding sites are shown in orange in the 3JQ4 template. (**bottom**) Several views of the amino acid substitutions are shown. (a) The residue highlighted in green shows the wild-type amino acid while the residue in light blue show the corresponding mutated residue at position 48 of the protein. (b,c) Different views of the amino acid side chains surrounding the V48M missense substitution.
(PDF)

S1 Table Details of five highest associated locations of SNPs to *CMAH* that remained genome-wide significant after permutation testing. (PDF)

S2 Table Details of the 5’ UTR deletion, c.139C>T (LVS only samples), c.142G>A and c.364C>T results in all samples with known serology. (XLSX)

S3 Table DNA SNP analyses of *CMAH* in type A and AB cats. (PDF)

S4 Table Details of VGL genotyping results for all the samples with unknown serology. (DOCX)

S5 Table Primers sequences for *CMAH* analysis and genotyping. (PDF)

## Abbreviations

BLAST: Basic Local Alignment Search Tool
CDS: coding sequence
CMAH: cytidine monophospho-N-acetylneuraminic acid hydroxylase
GWAS: genome-wide association study
LVS: Langford Veterinary Services
MDS: multi-dimensional scaling
PCR: polymerase chain reaction
PSI-BLAST: Position-Specific Iterated BLAST
SNP: single nucleotide polymorphism
Taq: Thermus aquaticus
UTR: untranslated region
VGL: Veterinary Genetics Laboratory
