# Correction: A Novel Variant in *CMAH* Is Associated with Blood Type AB in Ragdoll Cats

**DOI:** 10.1371/journal.pone.0194471

**Published:** 2018-03-13

**Authors:** Barbara Gandolfi, Robert A. Grahn, Nicholas A. Gustafson, Daniela Proverbio, Eva Spada, Badri Adhikari, Janling Cheng, Gordon Andrews, Leslie A. Lyons, Chris R. Helps

## Notice of republication

This article was republished on February 20, 2018, to correct an error where a previous version of S4 Table was published in error. Please download this article again to view the correct version.
