# Supplementary figures and images for: A Novel Variant in CMAH Is Associated with Blood Type AB in Ragdoll Cats
Source: PLoS One. 2016 May 12;11(5):e0154973. doi: 10.1371/journal.pone.0154973 (PMC4865243; doi:10.1371/journal.pone.0154973)

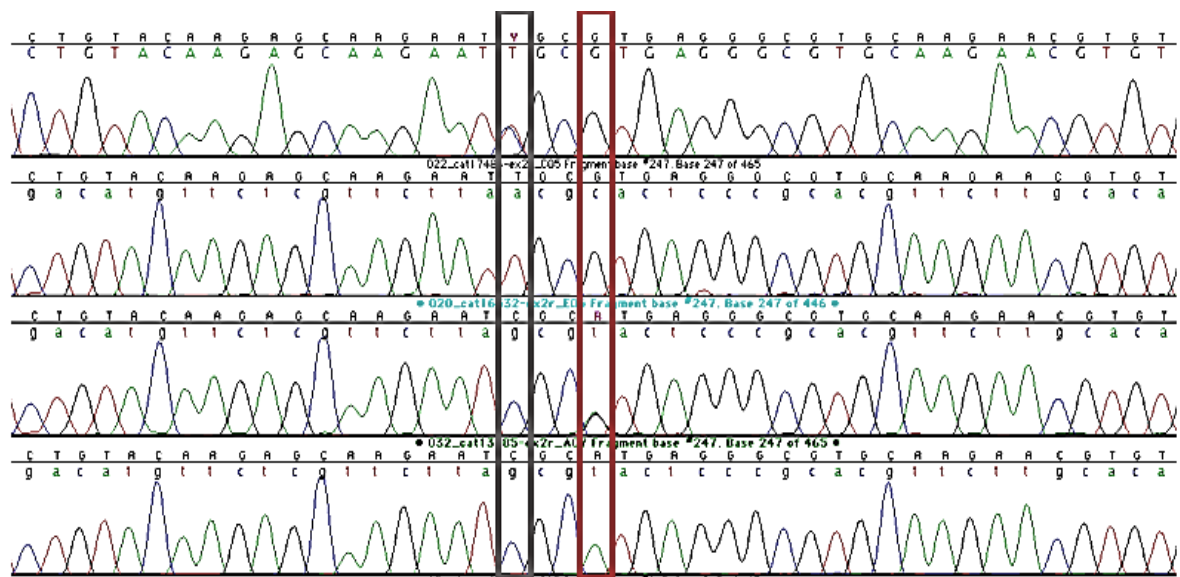

.a

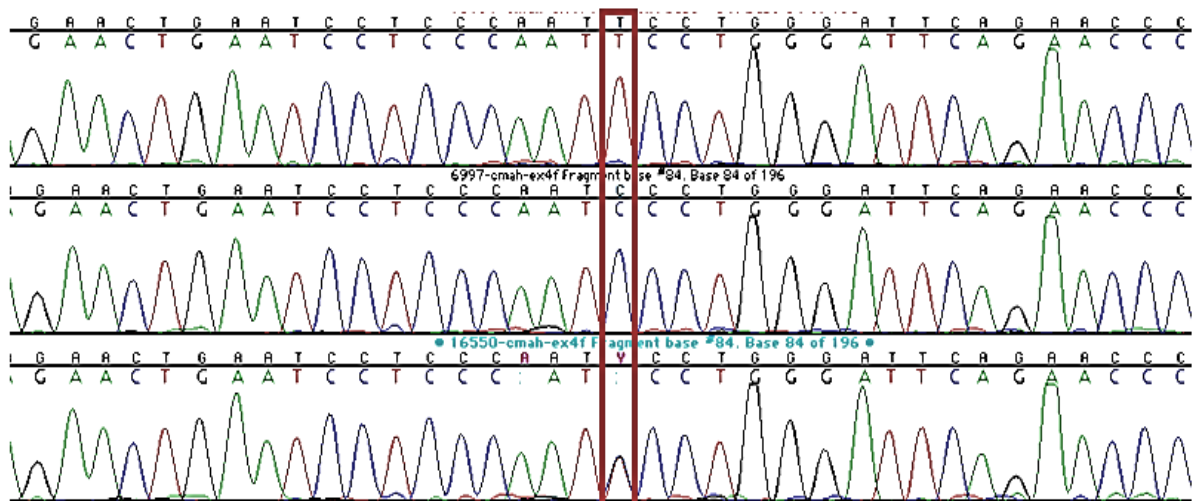

.b

Supplement: S1 Fig — a. The c.142G>A variant associated with blood type B (red rectangle), all possible genotypes are shown. Three bases upstream, in the black rectangle, is shown the c.139C>T variant. b. The c.364C>T variant associated with blood type AB (red rectangle). All possible genotypes are shown. (PDF) [file pone.0154973.s003.pdf]

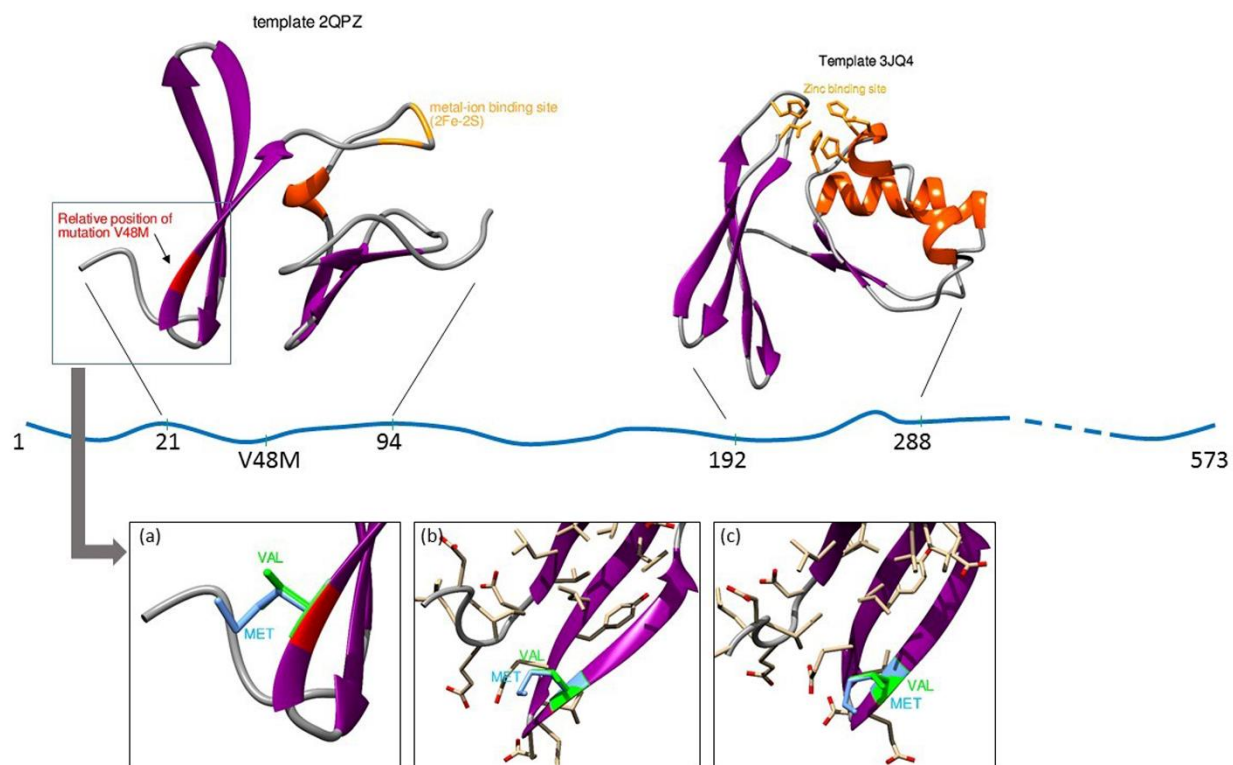

Supplement: S2 Fig — (top) The two domains found in the database used to partially modulate the structure of the protein from position 21 to position 94 and from position 192 to position 288 of the CMAH protein. The relative position of the V48M variant associated with blood type B is shown in red in the 2QPZ template and the four zinc-binding sites are shown in orange in the 3JQ4 template. (bottom) Several views of the amino acid substitutions are shown. (a) The residue highlighted in green shows the wild-type amino acid while the residue in light blue show the corresponding mutated residue at position 48 of the protein. (b,c) Different views of the amino acid side chains surrounding the V48M missense substitution. (PDF) [file pone.0154973.s004.pdf]
