# Supplementary material for: A Novel Variant in CMAH Is Associated with Blood Type AB in Ragdoll Cats
Source: PLoS One. 2016 May 12;11(5):e0154973. doi: 10.1371/journal.pone.0154973 (PMC4865243; doi:10.1371/journal.pone.0154973)
Supplement: S1 Table — (PDF) [file pone.0154973.s005.pdf]

S1 Table. Details of five highest associated locations of SNPs to *CMAH* that remained genome-wide significant after permutation testing.

Chromosome	SNP ID	6.2 assembly position	P_{raw}	P_{genome}
B2	chrB2.4545019	4487432	2.50e-10	0.00001
B2	chrB2.4625051	4449822	4.28e-5	0.004
B1	chrB1.122267584	99024960	8.10e-5	0.31
B1	chrB1.222834707	198985796	2.04e-5	0.56
C2	ChrC2.95368178	85312236	3.32e-5	0.71
