# Supplementary material for: A Novel Variant in CMAH Is Associated with Blood Type AB in Ragdoll Cats
Source: PLoS One. 2016 May 12;11(5):e0154973. doi: 10.1371/journal.pone.0154973 (PMC4865243; doi:10.1371/journal.pone.0154973)
Supplement: S3 Table — (PDF) [file pone.0154973.s007.pdf]

S3 Table. DNA SNP analyses of *CMAH* in type A and AB cats.

		Δ-53 18bp	E2 c.142	E3 c.268	I3 c.307 +22	E4 c.327	E7 c.884	I9 c.1112+29	I9 c.1112+66	I9 C.1113-70	E11 c.1269	E11 c.1398	E13 c.1603
Breed	Type	del	G>A	T>A	A>G	A>C	G>A	T>G	G>A	G>A	A>G	G>T	G>A
Devon rex	AB	NP	R	W	R	M	G	T	R	W	A	K	R
Devon rex	AB	NP	R	W	R	M	G	T	R	W	A	K	R
British SH	AB	NP	R	W	R	M	G	T	R	W	A	K	R
Random bred	AB	NP	R	W	R	C	R	K	G	W	A	K	R
Random bred	A	NN	G	T	R	A	G	T	R	A	R	G	G
Trace	A	NN	G	T	A	A	G	T	G	A	G	G	G
AA change			V48M	Y90N		P109S		-	-		-	-	D535N
