# Supplementary material for: A Novel Variant in CMAH Is Associated with Blood Type AB in Ragdoll Cats
Source: PLoS One. 2016 May 12;11(5):e0154973. doi: 10.1371/journal.pone.0154973 (PMC4865243; doi:10.1371/journal.pone.0154973)
Supplement: S4 Table — (DOCX) [file pone.0154973.s008.docx]

### S4 Table. Details of VGL genotyping results for all the samples with unknown serology.

| Case | Breed | Country | exon4 | indel | snp |
| --- | --- | --- | --- | --- | --- |
| FC-XAZDE | BG | US | N | AB | A |
| FC-WYBAA | BG |  | N | AB | A |
| FC-WWKKD | BG |  | N | AB | A |
| FC-WZBBZ | BS |  | N | B | AB |
| FC-WEWBW | BU |  | N | AB | A |
| FC-WEWBD | BU |  | N | AB | A |
| FC-WDCXY | BU |  | N | AB | A |
| FC-WBYWE | BU |  | N | AB | A |
| FC-WBEEZ | BU |  | N | AB | A |
| FC-WBEEY | BU |  | N | B | A |
| FC-XBBDB | DM | US | N | AB | A |
| FC-XBWAB | DM | US | N | AB | A |
| FC-XBYWX | DS | US | N | AB | A |
| FC-WWZZY | ES |  | N | AB | A |
| FC-WWYDB | ES |  | N | AB | A |
| FC-WDADC | ES |  | N | AB | A |
| FC-WDADB | ES |  | N | AB | A |
| FC-XBXYZ | MC | US | N | AB | A |
| FC-WEDZD | NF | SE | N | AB | A |
| FC-WXDZY | NF |  | N | AB | A |
| FC-WEDZD | NF |  | N | AB | A |
| FC-XAWEA | RD | US | N | B | AB |
| FC-XBABY | RD | US | N | AB | A |
| FC-XBABZ | RD | US | N | AB | A |
| FC-XBDDB | RD | US | N | AB | A |
| FC-XBWAA | RD | US | N | AB | A |
| FC-XBZEK | RD | US | N | AB | A |
| FC-XCKEZ | RD | US | NC | AB | A |
| FC-XCEKB | RD | US | NC | AB | A |
| FC-XKADC | RD |  | N | AB | A |
| FC-XKKZC | RD |  | N | AB | A |
| FC-WZZCZ | RD |  | N | B | AB |
| FC-WYYWY | RD |  | N | B | A |
| FC-WWXEK | RD |  | N | AB | A |
| FC-WEWWK | RD |  | N | AB | A |
| FC-WBDKC | RD |  | N | AB | A |
| FC-WBDKD | RD |  | NC | AB | A |
| FC-ECYBE | SC | NO | N | AB | A |
| FC-XBBZX | SC | SE | N | AB | A |
| FC-XBWYC | SC | SE | N | AB | A |
| FC-WZXZB | SC |  | N | AB | A |
| FC-XBKAB | SV | CA | N | AB | A |
| FC-WCAED | SV |  | N | AB | A |
| FC-XCEWE | SX | US | N | AB | A |
| FC-XCWDC | SX | NO | N | AB | A |
| FC-WXKBC | SX |  | N | AB | A |
| FC-WZCKW | TG |  | N | AB | A |
| FC-WZCKB | TG |  | N | B | AB |
| FC-WYZCE | TG |  | N | AB | A |
| FC-XAEYC | TO | US | N | AB | A |
| FC-XCEKD | UN | US | N | B | AB |
| FC-KKKKAY | UN | US | N | AB | A |
| FC-WDDAY | UN |  | N | B | AB |
| FC-CWDZE | AB | AU | N | A | A |
| FC-DKXKX | AB | JP | N | A | A |
| FC-WEBXW | AB | NL | N | AB | AB |
| FC-WEBXX | AB | NL | N | A | A |
| FC-XKABA | AB | SE | N | AB | AB |
| FC-XADYW | AB | DK | N | AB | AB |
| FC-XADYX | AB | DK | N | AB | AB |
| FC-XADYY | AB | DK | N | A | A |
| FC-XAEZA | AB | JP | N | AB | AB |
| FC-XAXAB | AB | RU | N | A | A |
| FC-XAXXD | AB | US | N | A | A |
| FC-XAXXE | AB | US | N | A | A |
| FC-XAYDX | AB | US | N | A | A |
| FC-XAYDY | AB | US | N | A | A |
| FC-XAZKB | AB | JP | N | A | A |
| FC-XAZKC | AB | JP | N | A | A |
| FC-XAZKD | AB | JP | N | AB | AB |
| FC-XBKAC | AB | BE | N | A | A |
| FC-XBAAZ | AB | CH | N | A | A |
| FC-XBADY | AB | DK | N | AB | AB |
| FC-XBDKK | AB | DE | N | AB | AB |
| FC-XBDKA | AB | DE | N | AB | AB |
| FC-XBDBK | AB | US | N | AB | AB |
| FC-XBEWX | AB | CH | N | A | A |
| FC-XBWAK | AB | BY | N | AB | AB |
| FC-XBWAD | AB | RU | N | A | A |
| FC-XBXCB | AB | JP | N | AB | AB |
| FC-XBXCC | AB | JP | N | A | A |
| FC-XBYYZ | AB | US | N | A | A |
| FC-XCAXW | AB | US | N | A | A |
| FC-XCBZZ | AB | RU | N | A | A |
| FC-XCDAK | AB | RU | N | AB | AB |
| FC-XCDAA | AB | RU | N | A | A |
| FC-XCDAB | AB | RU | N | AB | AB |
| FC-XCDDX | AB | IT | N | AB | AB |
| FC-XCEWW | AB | RU | N | A | A |
| FC-XCWCD | AB | US | N | A | A |
| FC-XCWCE | AB | US | N | A | A |
| FC-XCWCW | AB | US | N | A | A |
| FC-XCWCX | AB | US | N | A | A |
| CR-000922 | AB | FR | N | AB | AB |
| FC-XAZXB | AC | IT | N | AB | AB |
| FC-XCDKZ | AC | FR | N | AB | AB |
| FC-XADXB | AM | AU | N | AB | AB |
| FC-XADXC | AM | AU | N | AB | AB |
| FC-XAWKX | BA | AU | N | A | A |
| FC-XAWKY | BA | AU | N | A | A |
| FC-EYYKY | BG | CA | N | A | A |
| FC-EZZKY | BG | US | N | A | A |
| FC-WZBDK | BG | FR | N | A | A |
| FC-XADDZ | BG | FR | N | A | A |
| FC-XAXED | BG | NL | N | AB | AB |
| FC-XAXEE | BG | NL | N | A | A |
| FC-XBXKE | BG | BE | N | A | A |
| FC-XCWBW | BG | HU | N | A | A |
| FC-XCWBX | BG | HU | N | A | A |
| FC-XAWWB | BI | IT | N | A | A |
| FC-XAZYC | BI | IT | N | A | A |
| FC-XAZYD | BI | IT | N | A | A |
| FC-XBAKB | BI | SE | N | A | A |
| FC-XBAKE | BI | NO | N | B | B |
| FC-XBAKW | BI | NO | N | AB | AB |
| FC-XBABK | BI | DK | N | A | A |
| FC-XBABA | BI | DK | N | AB | AB |
| FC-XBAWK | BI | US | N | B | B |
| FC-XBAWA | BI | EE | N | A | A |
| FC-XBAXZ | BI | EE | N | A | A |
| FC-XBBKZ | BI | US | N | B | B |
| FC-XBBEZ | BI | SE | N | AB | AB |
| FC-XBBWK | BI | SE | N | AB | AB |
| FC-XBCKB | BI | US | N | A | A |
| FC-XBCZY | BI | GB | N | A | A |
| FC-XBDCX | BI | NO | N | B | B |
| FC-XBDXW | BI | GB | N | AB | AB |
| FC-XBDXX | BI | GB | N | A | A |
| FC-XBDXY | BI | GB | N | A | A |
| FC-XBDXZ | BI | SE | N | A | A |
| FC-XBDYE | BI | US | N | B | B |
| FC-XBWKZ | BI | US | N | AB | AB |
| FC-XBXYE | BI | IT | N | AB | AB |
| FC-XBZAD | BI | US | N | AB | AB |
| FC-XBZEC | BI | SE | N | AB | AB |
| FC-XBZYD | BI | US | N | AB | AB |
| FC-XBZYE | BI | US | N | A | A |
| FC-XBZYW | BI | US | N | A | A |
| FC-XCBYD | BI | IT | N | AB | AB |
| FC-XCCAB | BI | CA | N | AB | AB |
| FC-XCCAC | BI | CA | N | AB | AB |
| FC-XCCAD | BI | CA | N | A | A |
| FC-XCCAE | BI | CA | N | AB | AB |
| FC-XCCAW | BI | CA | N | AB | AB |
| FC-XCCDE | BI | IT | N | AB | AB |
| FC-XCDXC | BI | DK | N | A | A |
| FC-XCECZ | BI | US | N | A | A |
| FC-XCWCY | BI | US | N | AB | AB |
| FC-XAXXY | BL | US | N | A | A |
| FC-XAYBY | BL | NL | N | AB | AB |
| FC-XBEAW | BL | FR | N | A | A |
| FC-XBBEY | BM | NL | N | A | A |
| FC-XCBYW | BM | US | N | A | A |
| FC-XCDBY | BM | US | N | A | A |
| FC-XCDBZ | BM | US | N | A | A |
| FC-BWCAX | BS |  | N | AB | AB |
| FC-WKWCD | BS | ES | N | B | B |
| FC-XKCDW | BS | IT | N | A | A |
| FC-XADWD | BS | AU | N | B | B |
| FC-XADXK | BS | US | N | AB | AB |
| FC-XADXZ | BS | NL | N | B | B |
| FC-XAEKE | BS | IL | N | AB | AB |
| FC-XAEKW | BS | IL | N | A | A |
| FC-XAEKX | BS | IL | N | B | B |
| FC-XAEKY | BS | IL | N | AB | AB |
| FC-XAEKZ | BS | IL | N | AB | AB |
| FC-XAEAK | BS | IL | N | A | A |
| FC-XAEXX | BS | CH | N | A | A |
| FC-XAEXY | BS | CH | N | A | A |
| FC-XAEXZ | BS | CH | N | AB | AB |
| FC-XAEYK | BS | CH | N | A | A |
| FC-XAWKA | BS | US | N | AB | AB |
| FC-XAXKD | BS | CA | N | B | B |
| FC-XAXDX | BS | CZ | N | B | B |
| FC-XAXZX | BS | SE | N | B | B |
| FC-XAYEK | BS | GB | N | B | B |
| FC-XAZKK | BS | NZ | N | AB | AB |
| FC-XAZAD | BS | LT | N | B | B |
| FC-XAZAE | BS | LT | N | AB | AB |
| FC-XAZAW | BS | LT | N | B | B |
| FC-XAZCE | BS | LT | N | B | B |
| FC-XAZCX | BS | LT | N | B | B |
| FC-XAZCY | BS | LT | N | B | B |
| FC-XAZCZ | BS | LT | N | B | B |
| FC-XAZDK | BS | LT | N | B | B |
| FC-XAZXA | BS | NL | N | B | B |
| FC-XBKKX | BS | CZ | N | B | B |
| FC-XBKKY | BS | CZ | N | B | B |
| FC-XBKZY | BS | CZ | N | AB | AB |
| FC-XBAWB | BS | LV | N | AB | AB |
| FC-XBAWC | BS | LV | N | AB | AB |
| FC-XBAXD | BS | NO | N | AB | AB |
| FC-XBBKA | BS | NO | N | AB | AB |
| FC-XBBBK | BS | US | N | AB | AB |
| FC-XBBBB | BS | LV | N | AB | AB |
| FC-XBBBC | BS | LV | N | AB | AB |
| FC-XBBZY | BS | DK | N | B | B |
| FC-XBCBZ | BS | CH | N | A | A |
| FC-XBCCK | BS | DK | N | AB | AB |
| FC-XBCCA | BS | DK | N | AB | AB |
| FC-XBCWW | BS | IL | N | B | B |
| FC-XBCWX | BS | IL | N | AB | AB |
| FC-XBCWY | BS | IL | N | B | B |
| FC-XBCWZ | BS | IL | N | AB | AB |
| FC-XBDEZ | BS | IL | N | AB | AB |
| FC-XBDYK | BS | US | N | B | B |
| FC-XBDYA | BS | US | N | A | A |
| FC-XBDYB | BS | US | N | AB | AB |
| FC-XBDYC | BS | NL | N | AB | AB |
| FC-XBDYD | BS | NL | N | AB | AB |
| FC-XBEEE | BS | SE | N | AB | AB |
| FC-XBEEW | BS | SE | N | AB | AB |
| FC-XBEEX | BS | SE | N | B | B |
| FC-XBEEY | BS | SE | N | AB | AB |
| FC-XBEEZ | BS | SE | N | AB | AB |
| FC-XBEWK | BS | SE | N | AB | AB |
| FC-XBWWA | BS | ES | N | B | B |
| FC-XBWWC | BS | US | N | B | B |
| FC-XBWXC | BS | US | N | AB | AB |
| FC-XBWXX | BS | NL | N | A | A |
| FC-XBXCA | BS | PT | N | AB | AB |
| FC-XBXZZ | BS | IT | N | A | A |
| FC-XBYKZ | BS | SE | N | A | A |
| FC-XBYCX | BS | US | N | AB | AB |
| FC-XBYEX | BS | FR | N | AB | AB |
| FC-XBYEY | BS | PT | N | B | B |
| FC-XBYEZ | BS | PT | N | AB | AB |
| FC-XBYWK | BS | NZ | N | B | B |
| FC-XBYWA | BS | NZ | N | A | A |
| FC-XBYWW | BS | US | N | AB | AB |
| FC-XBZEW | BS | SE | N | AB | AB |
| FC-XBZEX | BS | SE | N | AB | AB |
| FC-XBZEY | BS | SE | N | AB | AB |
| FC-XBZEZ | BS | SE | N | A | A |
| FC-XBZWK | BS | SE | N | A | A |
| FC-XCKCC | BS | US | N | A | A |
| FC-XCBYX | BS | FR | N | A | A |
| FC-XCBYY | BS | US | N | AB | AB |
| FC-XCBYZ | BS | US | N | A | A |
| FC-XCBZK | BS | US | N | A | A |
| FC-XCDKC | BS | IL | N | AB | AB |
| FC-XCDAX | BS | FR | N | A | A |
| FC-XCDBD | BS | SG | N | AB | AB |
| FC-XCDBE | BS | SG | N | A | A |
| FC-XCDDY | BS | GB | N | AB | AB |
| FC-XCDZX | BS | NZ | N | AB | AB |
| FC-XCDZY | BS | NZ | N | B | B |
| FC-XCDZZ | BS | NZ | N | AB | AB |
| FC-XCEKW | BS | CZ | N | AB | AB |
| FC-XCEWY | BS | SE | N | B | B |
| FC-XCWCZ | BS | DE | N | A | A |
| FC-XBCZE | BU | US | N | AB | AB |
| FC-XADWY | CR | LT | N | AB | AB |
| FC-XAXKB | CR | US | N | A | A |
| FC-XAXKC | CR | US | N | B | B |
| FC-XAXXX | CR | NL | N | AB | AB |
| FC-XBAWD | CR | SE | N | A | A |
| FC-XBAWE | CR | SE | N | AB | AB |
| FC-XBBKE | CR | FI | N | AB | AB |
| FC-XBBKW | CR | FI | N | AB | AB |
| FC-XBBWA | CR | FR | N | B | B |
| FC-XBBZZ | CR | CA | N | B | B |
| FC-XBCKK | CR | CA | N | AB | AB |
| FC-XBCKA | CR | CA | N | AB | AB |
| FC-XBWYA | CR | SE | N | B | B |
| FC-XBXAA | CR | CA | N | AB | AB |
| FC-XBXDY | CR | DK | N | B | B |
| FC-XBXDZ | CR | DK | NC | A | A |
| FC-XBXYC | CR | BE | N | AB | AB |
| FC-XCABB | CR | FI | N | AB | AB |
| FC-XCAXX | CR | SE | N | AB | AB |
| FC-XCDCD | CR | SE | N | A | A |
| FC-XCDCE | CR | SE | N | A | A |
| FC-XAYXK | CX | US | N | A | A |
| FC-XAYXA | CX | US | N | B | B |
| FC-XAYXB | CX | US | N | AB | AB |
| FC-XAYXC | CX | US | N | A | A |
| FC-XBZYX | CX | US | N | AB | AB |
| FC-XAXDW | DL | US | N | A | A |
| FC-XBXWA | DM | US | N | A | A |
| FC-XCADA | DM | US | N | AB | AB |
| FC-WBDEC | DR | US | N | B | B |
| FC-XADCZ | DR | US | N | AB | AB |
| FC-XAEXE | DR | CH | N | AB | AB |
| FC-XAEXW | DR | CH | N | AB | AB |
| FC-XAEYA | DR | CH | N | A | A |
| FC-XAEYB | DR | CH | N | B | B |
| FC-XAWKZ | DR | US | N | A | A |
| FC-XAWBE | DR | RU | N | B | B |
| FC-XAWBW | DR | RU | N | AB | AB |
| FC-XAWBX | DR | RU | N | B | B |
| FC-XAYBE | DR | US | N | B | B |
| FC-XAYBW | DR | US | N | B | B |
| FC-XAYBX | DR | US | N | B | B |
| FC-XAYDZ | DR | SE | N | B | B |
| FC-XAYWD | DR | US | N | AB | AB |
| FC-XAYZE | DR | DE | N | B | B |
| FC-XAYZW | DR | DE | N | B | B |
| FC-XAZDA | DR | US | N | B | B |
| FC-XAZDB | DR | CA | N | AB | AB |
| FC-XBAYK | DR | CH | N | AB | AB |
| FC-XBAYA | DR | US | N | B | B |
| FC-XBBED | DR | US | N | AB | AB |
| FC-XBCKC | DR | SE | N | A | A |
| FC-XBDAZ | DR | NO | N | B | B |
| FC-XBDCY | DR | SE | N | B | B |
| FC-XBEWY | DR | DE | N | B | B |
| FC-XBEWZ | DR | DE | N | B | B |
| FC-XBWAW | DR | CA | N | AB | AB |
| FC-XBWAX | DR | CA | N | B | B |
| FC-XBWBK | DR | US | N | AB | AB |
| FC-XBWCA | DR | RU | N | AB | AB |
| FC-XBWXW | DR | SE | N | B | B |
| FC-XBWXZ | DR | US | N | AB | AB |
| FC-XBWYK | DR | US | N | B | B |
| FC-XBXWD | DR | IT | N | AB | AB |
| FC-XBXYB | DR | SE | N | AB | AB |
| FC-XBYZA | DR | US | N | A | A |
| FC-XCABA | DR | UA | N | AB | AB |
| FC-XCBKE | DR | US | N | AB | AB |
| FC-XCBKW | DR | US | N | AB | AB |
| FC-XCBKX | DR | US | N | AB | AB |
| FC-XCBWB | DR | US | N | B | B |
| FC-XCBXZ | DR | CH | N | AB | AB |
| FC-XCBYK | DR | CH | N | B | B |
| FC-XCBYA | DR | CH | N | B | B |
| FC-XCBYB | DR | CH | N | AB | AB |
| FC-XCBYC | DR | CH | N | AB | AB |
| FC-XCCAZ | DR | NO | N | B | B |
| FC-XCCBK | DR | NO | N | B | B |
| FC-XCCBA | DR | NO | N | AB | AB |
| FC-XCCBB | DR | NO | N | AB | AB |
| FC-XCCBC | DR | NO | N | B | B |
| FC-XCDAZ | DR | US | N | AB | AB |
| FC-XCDBK | DR | US | N | A | A |
| FC-XCDEX | DR | SE | N | A | A |
| FC-XCEDK | DR | US | N | B | B |
| FC-XAXWZ | DS | US | N | A | A |
| FC-XAZKA | DS | US | N | A | A |
| FC-XBAKX | DS | US | N | A | A |
| FC-XBXAD | DS | GB | N | AB | AB |
| FC-XBZXK | DS | US | N | AB | AB |
| FC-XCEKC | DS | US | N | AB | AB |
| CR-000919 | DS | US | NC | AB | AB |
| CR-000920 | DS | US | NC | AB | AB |
| CR-000921 | DS | US | NC | AB | AB |
| FC-XADXX | ES | US | N | A | A |
| FC-XADXY | ES | US | N | A | A |
| FC-XAXXZ | ES | US | N | A | A |
| FC-XAXYK | ES | US | N | A | A |
| FC-XBKED | ES | CA | N | AB | AB |
| FC-XBKZZ | ES | US | N | A | A |
| FC-XBAKK | ES | US | N | A | A |
| FC-XBAKA | ES | US | N | A | A |
| FC-XBAKD | ES | SE | N | A | A |
| FC-XBBDK | ES | IT | N | AB | AB |
| FC-XBBEE | ES | IT | N | A | A |
| FC-XBCZX | ES | RU | N | AB | AB |
| FC-XBDXD | ES | RU | N | A | A |
| FC-XBEAA | ES | SE | N | A | A |
| FC-XBEAE | ES | US | N | A | A |
| FC-XBYCK | ES | CA | N | A | A |
| FC-XBYCA | ES | CA | N | A | A |
| FC-XCABW | ES | US | N | A | A |
| FC-XCBCK | ES | LV | N | A | A |
| FC-XCBCA | ES | LV | N | A | A |
| FC-XCBCB | ES | LV | N | A | A |
| FC-WXZEY | KBL | DK | N | A | A |
| FC-XCADK | KM | US | N | AB | AB |
| FC-XBXYW | MC | US | N | A | A |
| FC-XBXYX | MC | US | N | A | A |
| FC-XBXYY | MC | US | N | A | A |
| FC-XBXZK | MC | US | N | A | A |
| FC-XBXZA | MC | US | N | A | A |
| FC-XBXZB | MC | US | N | A | A |
| FC-XBXZC | MC | US | N | A | A |
| FC-XBZWX | MC | US | N | A | A |
| FC-XBZWY | MC | US | N | B | B |
| FC-KKKKCK | MC | DE | N | A | A |
| FC-KKKKCC | MC | DE | N | AB | AB |
| FC-KKKKCD | MC | DE | N | A | A |
| FC-KKKDDY | MC | DE | N | A | A |
| FC-KKKDXX | MC | DE | N | A | A |
| FC-KKKDXY | MC | DE | N | AB | AB |
| FC-KKKDXZ | MC | DE | N | A | A |
| FC-KKKDYK | MC | DE | N | A | A |
| FC-KKKDYA | MC | DE | N | A | A |
| FC-KKKDYB | MC | DE | N | AB | AB |
| FC-KKKDYC | MC | DE | N | B | B |
| FC-XCKKZ | MX | FI | N | A | A |
| FC-WYXZZ | NF | DE | N | A | A |
| FC-XAKCX | NF | DE | N | A | A |
| FC-XBEBB | OC | SE | N | A | A |
| FC-XBKBW | PE | SE | N | B | B |
| FC-XBYZK | PE | FI | N | A | A |
| FC-WWKEW | RD | US | N | A | A |
| FC-WZECD | RD | US | N | A | A |
| FC-XKZBB | RD | US | N | A | A |
| FC-XADDW | RD | CZ | N | A | A |
| FC-XADXA | RD | US | N | A | A |
| FC-XADZX | RD | US | N | A | A |
| FC-XAEKC | RD | US | N | A | A |
| FC-XAEAA | RD | FR | N | A | A |
| FC-XAEAX | RD | AU | N | A | A |
| FC-XAEAY | RD | AU | N | A | A |
| FC-XAEAZ | RD | AU | N | A | A |
| FC-XAEBK | RD | CZ | N | A | A |
| FC-XAEBY | RD | US | NC | A | A |
| FC-XAEEZ | RD | AT | N | A | A |
| FC-XAEWK | RD | HU | N | A | A |
| FC-XAEWA | RD | HU | N | AB | AB |
| FC-XAEWB | RD | HU | N | A | A |
| FC-XAEWC | RD | US | N | A | A |
| FC-XAEWD | RD | US | N | A | A |
| FC-XAEWW | RD | FR | N | A | A |
| FC-XAEWX | RD | FR | N | A | A |
| FC-XAEYD | RD | US | N | A | A |
| FC-XAWCC | RD | US | N | A | A |
| FC-XAWCD | RD | US | N | A | A |
| FC-XAWCE | RD | US | N | A | A |
| FC-XAWCW | RD | US | N | A | A |
| FC-XAWEB | RD | US | NC | A | A |
| FC-XAWEC | RD | US | N | AB | AB |
| FC-XAWED | RD | US | N | A | A |
| FC-XAXAA | RD | US | NC | A | A |
| FC-XAXBB | RD | US | N | A | A |
| FC-XAXBD | RD | US | N | A | A |
| FC-XAYAC | RD | DK | N | A | A |
| FC-XAYAD | RD | US | N | A | A |
| FC-XAYAE | RD | US | N | A | A |
| FC-XAYAW | RD | US | N | A | A |
| FC-XAYAX | RD | US | N | A | A |
| FC-XAYAY | RD | CA | N | A | A |
| FC-XAYAZ | RD | CA | N | A | A |
| FC-XAYBK | RD | CA | N | A | A |
| FC-XAYBA | RD | CA | N | A | A |
| FC-XAYBB | RD | CA | N | A | A |
| FC-XAYBC | RD | CA | N | A | A |
| FC-XAYBD | RD | CA | N | A | A |
| FC-XAYCC | RD | SG | NC | A | A |
| FC-XAYCD | RD | SG | N | A | A |
| FC-XAYCE | RD | SG | NC | A | A |
| FC-XAYWX | RD | US | N | A | A |
| FC-XAYWY | RD | US | NC | A | A |
| FC-XAYWZ | RD | US | N | A | A |
| FC-XAZAC | RD | US | N | A | A |
| FC-XAZCD | RD | US | NC | AB | AB |
| FC-XAZXC | RD | CA | N | A | A |
| FC-XAZXD | RD | ES | N | A | A |
| FC-XBKKE | RD | IT | N | A | A |
| FC-XBKKW | RD | IT | NC | A | A |
| FC-XBKAY | RD | US | N | A | A |
| FC-XBKAZ | RD | US | N | A | A |
| FC-XBKBK | RD | US | N | A | A |
| FC-XBKBA | RD | US | N | A | A |
| FC-XBKBB | RD | US | N | AB | AB |
| FC-XBKDZ | RD | US | N | A | A |
| FC-XBKZW | RD | CZ | N | A | A |
| FC-XBKZX | RD | CZ | N | AB | AB |
| FC-XBAKC | RD | US | N | A | A |
| FC-XBACK | RD | US | N | AB | AB |
| FC-XBACA | RD | US | N | AB | AB |
| FC-XBACB | RD | US | NC | A | A |
| FC-XBACC | RD | US | NC | A | A |
| FC-XBACD | RD | US | NC | A | A |
| FC-XBAEZ | RD | US | NC | A | A |
| FC-XBAXA | RD | US | N | A | A |
| FC-XBAXE | RD | US | N | A | A |
| FC-XBAXW | RD | US | N | A | A |
| FC-XBAXX | RD | US | N | A | A |
| FC-XBAXY | RD | US | N | A | A |
| FC-XBBYZ | RD | CA | N | A | A |
| FC-XBCBA | RD | BE | NC | A | A |
| FC-XBCBB | RD | BE | NC | A | A |
| FC-XBCBC | RD | US | N | A | A |
| FC-XBCBD | RD | US | N | A | A |
| FC-XBCBY | RD | CA | N | A | A |
| FC-XBCCB | RD | ES | N | AB | AB |
| FC-XBCCC | RD | ES | N | AB | AB |
| FC-XBCYC | RD | US | NC | A | A |
| FC-XBCYD | RD | US | N | A | A |
| FC-XBCYE | RD | US | N | A | A |
| FC-XBCYW | RD | SK | N | A | A |
| FC-XBDBA | RD | JP | N | A | A |
| FC-XBDCZ | RD | US | NC | AB | AB |
| FC-XBDEY | RD | CA | NC | A | A |
| FC-XBDWB | RD | CA | NC | A | A |
| FC-XBEWB | RD | US | NC | AB | AB |
| FC-XBEWC | RD | US | N | AB | AB |
| FC-XBEWD | RD | US | N | AB | AB |
| FC-XBWAE | RD | US | NC | A | A |
| FC-XBWEZ | RD | US | N | A | A |
| FC-XBWWK | RD | CA | N | A | A |
| FC-XBWWB | RD | BE | N | A | A |
| FC-XBWXY | RD | CA | N | A | A |
| FC-XBWYD | RD | FR | N | A | A |
| FC-XBXAX | RD | US | N | A | A |
| FC-XBXBZ | RD | CA | N | A | A |
| FC-XBXCK | RD | CA | N | A | A |
| FC-XBXEK | RD | US | N | A | A |
| FC-XBXEA | RD | CA | N | A | A |
| FC-XBXWC | RD | CA | N | A | A |
| FC-XBXYK | RD | FR | N | A | A |
| FC-XBXYA | RD | FR | N | A | A |
| FC-XBXYD | RD | BE | N | A | A |
| FC-XBXZY | RD | US | N | B | B |
| FC-XBYCB | RD | US | N | A | A |
| FC-XBYCC | RD | US | N | A | A |
| FC-XBYCD | RD | US | NC | A | A |
| FC-XBYCE | RD | CA | N | A | A |
| FC-XBYCW | RD | CA | N | A | A |
| FC-XBYCY | RD | NL | N | A | A |
| FC-XBYYY | RD | US | C | A | A |
| FC-XBZAA | RD | US | N | A | A |
| FC-XBZAB | RD | US | N | A | A |
| FC-XBZAC | RD | US | N | A | A |
| FC-XBZDA | RD | CA | N | A | A |
| FC-XBZDW | RD | CA | NC | A | A |
| FC-XBZEA | RD | US | NC | A | A |
| FC-XBZEB | RD | US | N | A | A |
| FC-XBZYC | RD | US | N | A | A |
| FC-XCKAK | RD | US | NC | A | A |
| FC-XCKZK | RD | US | N | A | A |
| FC-XCKZA | RD | US | N | A | A |
| FC-XCBKY | RD | US | NC | A | A |
| FC-XCBKZ | RD | US | NC | A | A |
| FC-XCBCW | RD | BE | NC | A | A |
| FC-XCBYE | RD | US | N | A | A |
| FC-XCCBD | RD | US | N | A | A |
| FC-XCCBE | RD | US | N | A | A |
| FC-XCCBW | RD | US | N | A | A |
| FC-XCCBX | RD | US | N | A | A |
| FC-XCCCC | RD | US | N | A | A |
| FC-XCDAY | RD | FR | NC | A | A |
| FC-XCDEY | RD | US | N | A | A |
| FC-XCDXD | RD | US | NC | A | A |
| FC-XCEDB | RD | US | NC | A | A |
| FC-XCEDC | RD | US | N | A | A |
| FC-XCEDX | RD | CA | N | AB | AB |
| FC-XCEWZ | RD | US | N | A | A |
| FC-XCEXK | RD | US | N | AB | AB |
| FC-XCWAD | RD | US | N | A | A |
| FC-XCWAE | RD | US | NC | A | A |
| FC-XAXKE | RM | US | N | A | A |
| FC-XAXYA | RM | US | N | A | A |
| FC-XAYWW | RM | US | N | A | A |
| FC-XBEZK | RM | US | N | A | A |
| FC-XBEZA | RM | US | N | A | A |
| FC-XBZED | RM | US | N | A | A |
| FC-XBZEE | RM | US | N | AB | AB |
| FC-XCDCW | RM | US | N | AB | AB |
| FC-XCDCX | RM | US | N | AB | AB |
| FC-BXBCX | SC |  | N | AB | AB |
| FC-CEZBE | SC | NO | N | AB | AB |
| FC-CXKAB | SC | NO | N | A | A |
| FC-EAEWX | SC | NO | N | A | A |
| FC-EXEYZ | SC | NO | N | A | A |
| FC-EYWWY | SC | FR | N | A | A |
| FC-WKDZZ | SC | SE | N | A | A |
| FC-WYEKZ | SC | NO | N | A | A |
| FC-WYXKW | SC | NO | N | A | A |
| FC-WZCXK | SC | FR | N | A | A |
| FC-XADYZ | SC | CO | N | A | A |
| FC-XADZK | SC | CO | N | AB | AB |
| FC-XADZA | SC | CO | N | A | A |
| FC-XADZB | SC | CO | N | A | A |
| FC-XAEAB | SC | SE | N | A | A |
| FC-XAWAA | SC | ES | N | A | A |
| FC-XAWAB | SC | ES | N | AB | AB |
| FC-XAXKA | SC | SE | N | A | A |
| FC-XAXKX | SC | SE | N | A | A |
| FC-XBKBC | SC | NO | N | AB | AB |
| FC-XBKBD | SC | NO | N | A | A |
| FC-XBKBE | SC | NO | N | A | A |
| FC-XBCKD | SC | SE | N | A | A |
| FC-XBCWB | SC | SE | N | A | A |
| FC-XBDXE | SC | NO | N | A | A |
| FC-XBXAC | SC | GB | N | A | A |
| FC-XBXXX | SC | SE | N | A | A |
| FC-XBXXY | SC | SE | N | A | A |
| FC-XBXXZ | SC | SE | N | A | A |
| FC-XCKEY | SC | SE | N | A | A |
| FC-XCAXY | SC | NO | N | AB | AB |
| FC-XCAXZ | SC | NO | N | A | A |
| FC-XCEYC | SC | SE | N | A | A |
| FC-XCWCC | SC | NO | N | A | A |
| FC-XAWKB | SF | US | N | AB | AB |
| FC-XAWAK | SF | US | N | A | A |
| FC-XAXEB | SF | UA | N | B | B |
| FC-XAYXD | SF | US | N | A | A |
| FC-XBWAZ | SF | CA | N | A | A |
| FC-XCDBW | SF | SG | N | A | A |
| FC-XCDBX | SF | SG | N | A | A |
| FC-DCZDB | SI | US | N | AB | AB |
| FC-WKYZX | SI | US | N | A | A |
| FC-WABEZ | SI | US | N | A | A |
| CR KKKZBZ | SN |  | N | A | A |
| FC-XADWZ | SO | NO | N | A | A |
| FC-XAWBY | SO | DE | N | AB | AB |
| FC-XAWBZ | SO | DE | N | AB | AB |
| FC-XAWCK | SO | DE | N | AB | AB |
| FC-XAWCA | SO | DE | N | AB | AB |
| FC-XAWCB | SO | DE | N | AB | AB |
| FC-XAWZC | SO | JP | N | A | A |
| FC-XAWZD | SO | JP | N | AB | AB |
| FC-XAYYB | SO | US | N | A | A |
| FC-XBKEY | SO | JP | N | AB | AB |
| FC-XBKEZ | SO | JP | N | A | A |
| FC-XBKWK | SO | JP | N | A | A |
| FC-XBAAX | SO | CH | N | AB | AB |
| FC-XBCCE | SO | DE | N | AB | AB |
| FC-XBCCW | SO | DE | N | AB | AB |
| FC-XBCCX | SO | DE | N | AB | AB |
| FC-XBCZZ | SO | DE | N | AB | AB |
| FC-XBEXA | SO | SE | N | AB | AB |
| FC-XCKCD | SO | CA | N | AB | AB |
| FC-XCKCE | SO | CA | N | AB | AB |
| FC-XCKCX | SO | CA | N | AB | AB |
| FC-XCBWY | SO | DE | N | AB | AB |
| FC-XCDCC | SO | DE | N | AB | AB |
| FC-XCDEZ | SO | US | N | B | B |
| FC-XKZXX | SR | CA | N | A | A |
| FC-XAXKK | SR | DK | N | AB | AB |
| FC-XAXEY | SR | US | N | A | A |
| FC-XAXEZ | SR | US | N | A | A |
| FC-XAZAX | SX | BE | N | A | A |
| FC-XAZAY | SX | BE | N | A | A |
| FC-XAZAZ | SX | BE | N | AB | AB |
| FC-XAZBK | SX | BE | N | A | A |
| FC-XAZBA | SX | BE | N | AB | AB |
| FC-XBABB | SX | SE | N | AB | AB |
| FC-XBWYB | SX | FR | N | A | A |
| FC-XCABK | SX | SE | N | A | A |
| FC-XCAXE | SX | FR | N | A | A |
| FC-XCBAW | SX | NL | N | A | A |
| FC-XCBZC | SX | NO | N | A | A |
| FC-XCEAZ | SX | SE | N | AB | AB |
| FC-XCWDB | SX | NO | N | B | B |
| FC-XBCXK | TV | US | N | A | A |
| FC-XBYBK | TV | GB | N | A | A |
| FC-XABDD | UN | US | NC | AB | AB |
| FC-XADCX | UN | US | N | A | A |
| FC-XAXBE | UN | US | N | A | A |
| FC-XAYCX | UN | US | N | A | A |
| FC-XAYYK | UN | CA | N | AB | AB |
| FC-XAYYA | UN | US | N | B | B |
| FC-XAZXK | UN | US | N | A | A |
| FC-XBKDE | UN | US | N | A | A |
| FC-XBBKX | UN | US | N | A | A |
| FC-XBBEX | UN | US | NC | A | A |
| FC-XBCEC | UN | US | N | A | A |
| FC-XBCXA | UN | US | N | A | A |
| FC-XBEAB | UN | US | N | A | A |
| FC-XBXWK | UN | US | N | A | A |
| FC-XBZWW | UN | US | N | A | A |
| FC-XBZWZ | UN | US | N | A | A |
| FC-XCKYC | UN |  | N | A | A |
| FC-XCKYD | UN | US | NC | AB |  |
| FC-XCACZ | UN | US | N | A | A |
| FC-KKKKKX | UN | US | N | A | A |
| FC-KKKKKY | UN | US | N | A | A |
| FC-KKKKKZ | UN | US | N | AB | AB |
| FC-KKKKAK | UN | US | N | AB | AB |
| FC-KKKKAA | UN | US | N | B | B |
| FC-KKKKAB | UN | US | N | A | A |
| FC-KKKKAC | UN | US | NC | A | A |
| FC-KKKKAD | UN | US | N | A | A |
| FC-KKKKAE | UN | US | N | A | A |
| FC-KKKKAW | UN | US | N | A | A |
| FC-KKKKAX | UN | US | N | AB | AB |
| FC-KKKKAZ | UN | US | N | A | A |
| FC-KKKKBK | UN | US | N | AB | AB |
| FC-KKKKBA | UN | US | N | A | A |
| FC-KKKKBB | UN | US | N | AB | AB |
| FC-XKXZX | RD |  | N | A | A |
| FC-XKXZZ | RD |  | N | A | A |
| FC-XKYKK | RD |  | N | A | A |
| FC-XKYKA | RD |  | N | A | A |
| FC-XKWWX | RD |  | N | B | B |
| FC-XKEAD | RD |  | N | A | A |
| FC-XKCWB | RD |  | N | A | A |
| FC-XKBEB | RD |  | N | A | A |
| FC-XKBDB | RD |  | N | A | A |
| FC-XKBKE | RD |  | N | A | A |
| FC-XKBKC | RD |  | N | A | A |
| FC-XKBKB | RD |  | N | A | A |
| FC-XKBKA | RD |  | N | A | A |
| FC-XKBKK | RD |  | N | A | A |
| FC-XKAZZ | RD |  | N | A | A |
| FC-XKAZX | RD |  | N | A | A |
| FC-XKKZX | RD |  | N | A | A |
| FC-XKKZE | RD |  | N | A | A |
| FC-XKKZB | RD |  | N | A | A |
| FC-XKKAZ | RD |  | N | A | A |
| FC-XKKAY | RD |  | N | A | A |
| FC-XKKAX | RD |  | N | A | A |
| FC-XKKAW | RD |  | N | A | A |
| FC-XKKAE | RD |  | N | A | A |
| FC-WZZZE | RD |  | N | A | A |
| FC-WZZYE | RD |  | N | A | A |
| FC-WZZYD | RD |  | N | A | A |
| FC-WZZXY | RD |  | N | A | A |
| FC-WZZXX | RD |  | N | A | A |
| FC-WZZXW | RD |  | N | A | A |
| FC-WZZXD | RD |  | N | A | A |
| FC-WZZXC | RD |  | N | A | A |
| FC-WZZDC | RD |  | N | A | A |
| FC-WZZDK | RD |  | N | A | A |
| FC-WZZCD | RD |  | N | A | A |
| FC-WZZCC | RD |  | N | A | A |
| FC-WZZCB | RD |  | N | A | A |
| FC-WZZBC | RD |  | N | A | A |
| FC-WZZBB | RD |  | N | A | A |
| FC-WZYKE | RD |  | N | A | A |
| FC-WZXZE | RD |  | N | A | A |
| FC-WZXZD | RD |  | N | A | A |
| FC-WZXZC | RD |  | N | A | A |
| FC-WZXXB | RD |  | N | A | A |
| FC-WZWDZ | RD |  | N | A | A |
| FC-WZECE | RD |  | N | A | A |
| FC-WZECD | RD |  | N | A | A |
| FC-WZECC | RD |  | N | A | A |
| FC-WZDCZ | RD |  | N | A | A |
| FC-WZDCY | RD |  | N | A | A |
| FC-WZDCX | RD |  | N | A | A |
| FC-WZDCW | RD |  | N | A | A |
| FC-WZDBD | RD |  | N | A | A |
| FC-WZDBC | RD |  | N | A | A |
| FC-WZDBB | RD |  | N | A | A |
| FC-WZDKB | RD |  | N | A | A |
| FC-WZDKA | RD |  | N | A | A |
| FC-WZDKK | RD |  | N | A | A |
| FC-WZCZY | RD |  | N | A | A |
| FC-WZCZX | RD |  | N | A | A |
| FC-WZCBW | RD |  | N | A | A |
| FC-WZCBA | RD |  | N | A | A |
| FC-WZCAZ | RD |  | N | A | A |
| FC-WZBZE | RD |  | N | A | A |
| FC-WZBZD | RD |  | N | A | A |
| FC-WZBEY | RD |  | N | A | A |
| FC-WZBEW | RD |  | N | A | A |
| FC-WZBED | RD |  | N | A | A |
| FC-WZBEC | RD |  | N | A | A |
| FC-WZBEB | RD |  | N | A | A |
| FC-WZBEA | RD |  | N | A | A |
| FC-WZBEK | RD |  | N | A | A |
| FC-WZBDZ | RD |  | N | A | A |
| FC-WZBDY | RD |  | N | A | A |
| FC-WZBDX | RD |  | N | A | A |
| FC-WZBKC | RD |  | N | A | A |
| FC-WZAXZ | RD |  | N | A | A |
| FC-WZAXY | RD |  | N | A | A |
| FC-WZAXX | RD |  | N | A | A |
| FC-WZAXW | RD |  | N | A | A |
| FC-WZADX | RD |  | N | A | A |
| FC-WZADW | RD |  | N | A | A |
| FC-WZACY | RD |  | N | A | A |
| FC-WZKYD | RD |  | N | A | A |
| FC-WZKYC | RD |  | N | A | A |
| FC-WZKYB | RD |  | N | A | A |
| FC-WZKCY | RD |  | N | A | A |
| FC-WZKCW | RD |  | N | A | A |
| FC-WZKCE | RD |  | N | A | A |
| FC-WYZZB | RD |  | N | A | A |
| FC-WYZCC | RD |  | N | A | A |
| FC-XKXZY | RD |  | NC | A | A |
| FC-XKKZY | RD |  | NC | A | A |
| FC-XKKZW | RD |  | NC | A | A |
| FC-WZZDA | RD |  | NC | A | A |
| FC-WZDKC | RD |  | NC | A | A |
| FC-WZCZZ | RD |  | NC | A | A |
| FC-WZBEX | RD |  | NC | A | A |
| FC-WZBKD | RD |  | NC | A | A |
| FC-WZAYK | RD |  | NC | A | A |
| FC-WZACK | RD |  | NC | A | A |
| FC-WZAAX | RD |  | NC | A | A |
| FC-XKXZA | AB |  | N | B | B |
| FC-XKWEA | AM |  | N | B | B |
| FC-XKXZK | BG |  | N | A | A |
| FC-XKXXA | BI |  | N | AB | AB |
| FC-XKXXB | BI |  | N | A | A |
| FC-XKXXC | BI |  | N | A | A |
| FC-XKXXD | BI |  | N | AB | AB |
| FC-WEWBE | BU |  | N | B | A |
| FC-XKWWW | DR |  | N | B | B |
| FC-XKWWX | DR |  | N | B | B |
