# Supplementary material for: A Novel Variant in CMAH Is Associated with Blood Type AB in Ragdoll Cats
Source: PLoS One. 2016 May 12;11(5):e0154973. doi: 10.1371/journal.pone.0154973 (PMC4865243; doi:10.1371/journal.pone.0154973)
Supplement: S5 Table — (PDF) [file pone.0154973.s009.pdf]

S5 Table. Primers sequences for *CMAH* analysis and genotyping.

	Genomic analysis	
CMAH region	Forward primer	Reverse primer
Exon 1	ttcctttgctgctggagtg	gctctgcttgctcsgtgttg
Exon 2*	tgagcaagcagagcgtgcat	ctaagtgagcacacacgaagattatgaatt
Exon 3	ggtgctcaagcccctcatttaa	tggtcacagtcttgtcctaggcac
Exon 4$	ccctttgatttttggtactctgtgtac	taatacacacacataaatggacatcttag
Exon 5	taaaaccaaccgggagagtttctat	acacatgaaaactgtatgatattcgca
Exon 6-7	ggagcaaactgtctcttctactaggtga	gaaatgaagatagtagaagatggttatcactc
Exon 8	ttccttggatagaagtaattcttctctgat	gtttcccagttaagactgtaaacttcttg
Exon 9	agagagcacttttcactcatgacaca	agacaatctctctgaatggattctagct
Exon 10	aagacaaagtataagtacaacttcttggactaa	acaaagacttaatattcctacaaacgagg
Exon 11	caaaagatgtggagttttcccatagt	aacaatcgtgggacaagaatgct
Exon 12	gagggactatacgaactccaattcatt	gacaactagcacagtaaaggaaggtg
Exon 13	tgcgttctgtagatgaggagactgc	gaatgaagctctaatggtggaatctca
Exon 14	aaatgccaggcactgaaacagta	gatttagctctgaacactcactgcc

Primer name	*RNA analysis*	
CMAH-5UTR		tgacagaccttccgtctaaatcttc
CMAH-1F	atgctcctttgagttcccttgact	
CMAH-2F	gagcaccatgaaatacgtcaatcc	
CMAH-3F	tcgaatatggcagcaggtaga	
CMAH-4F	aaatgacccaaatgaactcaataatctt	
CMAH-3UTR	ggacgacttgtatataggattccaaac	
CMAH-1R		cattttcttcatccatttcaacaactag
CMAH-2R		agtccactgtattaagtatcttatgaccttt
CMAH-3R		tctatgatgcccttgctgtctgt
CMAH-4R		aaaacaggtgatggtatatgtcaggac

Primer name	*Genotyping*	
FAM-CMAHdelF	tgagcaaaaggatcagatctaaacg	
CMAHdelR		aaatgcacgctctgcttgct
FAM-364F	FAM-cgtgtttgttgtgaatacgtg	
364wtR		tgggttctgaatccAaggg
364affectedR		gtttctttgggttctgaatcTcagga
CMAH ex2 F	Biotin-gaagaccggcaaagatttcat	
CMAH ex2 R		ctccttgatgcttgcacac
CMAH ex2 Seq		acacgttcttgcacgccctca
CMAH ex4 F	gaccctttggttccagttgttg	
CMAH ex4 R		Biotin-cacttctccaaaagccaaatgtt
CMAH ex4 Seq	cttttacttctagaactgaatcctcccaat	

*Primers used at the University of Missouri to genotype c.142G>A variant. $Primers used at the University of Missouri to genotype c.364C>T variant.
